# Succinylcholine-Induced Masseter Muscle Rigidity Successfully Managed With Propofol and Laryngeal Mask Airway: A Case Report and Brief Review

**DOI:** 10.7759/cureus.9376

**Published:** 2020-07-24

**Authors:** Muhammad Mubbashir Sheikh, Adeel Riaz, Hafiz M Umair, Muhammad Waqar, Ahmad Muneeb

**Affiliations:** 1 Oncology, Northwestern University Feinberg School of Medicine, Chicago, USA; 2 Anesthesiology and Critical Care, District Headquarter Hospital, Sahiwal, PAK; 3 Anaesthesia, District Headquarter Teaching Hospital, Sahiwal, PAK; 4 Anesthesia and Critical Care, District Headquarter Teaching Hospital, Sahiwal, PAK; 5 Internal Medicine, Allied Hospital / Faisalabad Medical University, Faisalabad, PAK

**Keywords:** masseter muscle spasm, malignant hyperthermia, succinylcholine, difficult airway, propofol, laryngeal mask airway

## Abstract

Masseter muscle rigidity following administration of succinylcholine for induction of general anesthesia is considered an early warning sign for the possibility of an episode of dreaded complication i.e., malignant hyperthermia. This report describes a case of masseter muscle rigidity encountered at the start of an emergency surgical procedure. After succinylcholine administration, laryngoscopy and intubation were not possible due to the masseter muscle spasms. This led to the use of laryngeal mask airway and propofol for the successful conclusion of the procedure with no intraoperative or postoperative complications. Later, the patient was discharged with instructions to avoid the contributing triggers in the future and recommendations of caffeine-muscle biopsy.

## Introduction

Succinylcholine is a short-acting, depolarizing skeletal muscle relaxant, that is widely used for endotracheal intubation because of its better muscle relaxation and rapid onset/offset profile [1]. The most common reported adverse effects are masseter muscle rigidity (MMR), malignant hyperthermia (MH), hypotension, and rarely hyperkalemia. MMR following succinylcholine administration has been reported as a rare isolated event, as described in this case as well [2]. Closed differentials like MH, tetanus (prevalent in Pakistan), and temporomandibular joint (TMJ) dysfunction should be kept in mind and ruled out if such difficulty arises [3]. This case highlights the role of laryngeal mask airway (LMA) that can be used as an alternative to secure airway if endotracheal intubation is not possible. Surgery can be conducted in a convenient way using total intravenous anesthetics (TIVA) without further exposure to halogenated agents.

## Case presentation

A 55-year-old, 68 kg male with no significant past medical history and surgical history presented to the emergency room for the emergent repair of obstructed umbilical hernia. Clinical examination and laboratory evaluation were in normal range except for a mild increase in blood urea nitrogen (BUN). The patient was classified as American Society of Anesthesiologists (ASA) class 2. Initially, he was managed conservatively and later shifted to the operating room after a fast of 6 hours. He denied a history of previous exposure to anesthetic agents and preoperative evaluation did not reveal any features suggestive of difficult intubation. Ringer lactate infusion was done via 18G IV cannula and premedicated with nalbuphine. Monitoring included electrocardiography (EKG/ECG), noninvasive blood pressure (NIBP), and pulse oximetry (SpO2). Baseline SpO2 was 98% on room air with a blood pressure of 135/92 and a heart rate of 108 beats per minute, respectively. General anesthesia (GA) was planned. The patient was pre-oxygenated with 100% oxygen for 5 minutes, and induction was performed using propofol 10 mg in incremental doses and succinylcholine (100 mg). After a minute of observing no fasciculations, laryngoscopy was attempted but failed due to the inability to open the mouth. His jaw was fixed, absolutely immobile (Jaws of steel) that made laryngoscopy impossible. Considering the risk of masseter muscle spasm, intubation was aborted and mask ventilation was started. Mask ventilation maintained oxygen saturation at 100%. The patient regained spontaneous respiration after 10 minutes abandoning external ventilation. Meanwhile, the temperature was checked and found to be 98°F. His musculoskeletal examination did not reveal any rigidity or stiffness anywhere else in the body. Urine output was also adequate and non-bloody.

Keeping in view high susceptibility of malignant hyperthermia (MH) and unavailability of life-saving drug dantrolene at our center, all halogen-containing anesthetics were withheld and the decision was changed from GA in favor of total intravenous anesthesia (TIVA). He was assessed again for mouth opening; jaw was a bit lax but still unable to perform laryngoscopy. Laryngeal mask airway (LMA) was possible and placed. Propofol was used for anesthesia maintenance for its demonstrated benefits and atracurium for muscle relaxation. Intraoperative vital monitoring remained unremarkable during the entire procedure. Neostigmine 2.5 mg and atropine 1 mg were used as reversal agents. The patient regained spontaneous respiration with adequate breathing effort and tidal volume. LMA was removed and SpO2 maintained at 98% on room air. His muscle power was normal with a head lift for more than 5 seconds. His mouth opening was now possible without any difficulty, returning to the baseline.

Post-operatively, the patient was upgraded to the intensive care unit (ICU) for closed observation of MH. Continuous vital signs monitoring was done for 24 hours and the patient remained afebrile, throughout. Laboratory evaluations were significant for creatine phosphokinase (CPK) levels of 889 U/L (normal range: 22-198 U/L) and serum potassium (K+) levels of 5.7 mmol/L (normal range: 3.5-5.2 mmol/L) with normal serum chemistry, hematology, and urinalysis. Absence of rigidity/stiffness anywhere else, normal body temperature, and elevated CPK; a diagnosis of isolated masseter muscle rigidity induced by succinylcholine was made and the patient was downgraded to floor. The patient's postoperative period was uneventful and was discharged on the third postoperative day with special instructions of not receiving succinylcholine or other halogenated anesthetics in the future. A muscle biopsy with caffeine testing was advised to rule out MH.

## Discussion

MMR following succinylcholine has an incidence of <1% in the pediatric population but a rare event in adults [4]. Classically, the term ‘Jaw of steel’ has been used for MMR that occurs with increased tension of jaw muscles. MMR can be an independent occurrence or it can be an early manifestation of MH. MH is the most feared complication as it is invariably fatal if not managed in time [5]. Table 1 shows different anesthetic agents that can trigger MH [1]. These triggers must be avoided if MMR develops.

**Table 1:** Most common triggers for malignant hyperthermia

General Anesthetics Agents	Depolarizing Muscle Relaxants
Halothane	Succinylcholine
Sevoflurane	
Enflurane	
Desflurane	
Isoflurane	

Succinylcholine induces muscle paralysis after a transient increase in muscle tone. This increased muscle tone, especially involving the jaw muscles, can persist in certain genetically susceptible patients, which may cause the inability to proceed with laryngoscopy and endotracheal intubation due to difficulty in mouth opening [1]. This clinical dilemma should be addressed promptly as a failure of intubation might cause significant morbidity and mortality. Endotracheal intubation can be attempted first but if impossible alternative ways to secure airway like LMA, nasotracheal intubation, or retrograde endotracheal intubation may be utilized per the availability. Surgical cricothyroidotomy and tracheostomy can be engaged as a last resort [6]. The use of LMA as a safe method to secure the airway is well documented [5]. In our patient, laryngoscopy was impossible so bag-mask ventilation was started to buy time. As soon as there was some laxity of the jaw, LMA was introduced to secure the airway.

Earlier it was suggested that surgery should be abandoned if MMR develops but there have been a few case reports of proceeding with the procedure using IV agents like thiopental, propofol, and non-depolarizing agents with careful monitoring [7]. Propofol is the anesthetic agent of choice for its multiple benefits in such situations. It can be used as an alternative for maintenance of anesthesia, helps relieve the rigidity of the jaw muscles, and acts as an anti-trigger agent of MH [8].

After completion of the procedure, the patient should be kept under observation in ICU for close monitoring of MH signs (high temperatures and severe muscle rigidity); abnormality in any specific parameters like arterial blood gases (ABGs), oxygen saturation, end-tidal CO2 (etCO2), and serum K+ levels, respectively [9]. Succinylcholine can cause a minute increase in serum K+ and CPK levels [10,11]. Our patient’s etCO2, ABGs, temperature, urinalysis remained unremarkable, and there was only a moderate increase in serum potassium and CPK levels confirming the diagnosis of isolated MMR. In short, this article addresses that when faced with an isolated MMR event with an uneventful intraoperative and postoperative period, such patients should be counseled to avoid triggering drugs in the future. “No Succinylcholine” should be mentioned in their medical record and muscle biopsy with caffeine testing must be done to rule out MH [12].

## Conclusions

This case report highlights MMR as an isolated event that can lead to failed laryngoscopy and endotracheal intubation. Airway must be secured quickly by any convenient means and vital monitoring should be done vigilantly both intraoperatively and postoperatively keeping the possibility of MH in mind. Muscle biopsy with caffeine testing should be stressed to rule out MH. Also, proper education and counseling should be provided related to the risks of triggering drugs in the future.

## References

[1] Bevan DR (1994). Succinylcholine. Can J Anaesth.

[2] Sharma M, Sharma S, Kalra P, Kulshreshtha A (2013). Succinylcholine induced masseter muscle rigidity: an isolated event. Anaesth Pain Intensive Care.

[3] Yemen TA (1993). Are we obsessed with masseter muscle rigidity? Temporomandibular joint disease mistakenly diagnosed as masseter muscle rigidity on two separate occasions in one patient. Anesth Analg.

[4] Lazzell VA, Carr AS, Lerman J, Burrows FA, Creighton RE (1994). The incidence of masseter muscle rigidity after succinylcholine in infants and children. Can J Anaesth.

[5] Onyeka TC (2010). Masseter muscle rigidity: atypical malignant hyperthermia presentation or isolated event?. Saudi J Anaesth.

[6] Bauer SJ, Orio K, Adams BD (2005). Succinylcholine induced masseter spasm during rapid sequence intubation may require a surgical airway: case report. Emerg Med J.

[7] Rosenberg H (1987). Trismus is not trivial. Anesthesiology.

[8] Ummenhofer WC, Kindler C, Tschalèr G, Hampl KF, Drewe J, Urwyler A (1998). Propofol reduces succinylcholine induced increase of masseter muscle tone. Can J Anaesth.

[9] Fitzpatrick LR (2008). Masseter muscle rigidity, elevated creatine kinase, and rhabdomyolysis following succinylcholine administration: a case report. AANA J.

[10] Rosenberg H, Pollock N, Schiemann A, Bulger T, Stowell K (2015). Malignant hyperthermia: a review. Orphanet J Rare Dis.

[11] Gronert GA (2009). Succinylcholine-induced hyperkalemia and beyond. Anesthesiology.

[12] The European Malignant Hyperpyrexia Group (1984). A protocol for the investigation of malignant hyperpyrexia (MH) susceptibility. Br J Anaesth.

